# Characteristics of Bulletproof Vests Made from COPEFB Fiber: Implications on Mechanical, Electrical, and Physical Resistance

**DOI:** 10.1155/2023/9475956

**Published:** 2023-05-31

**Authors:** Siti Nikmatin, Irmansyah Irmansyah, Endah Kinarya Palupi, Rofiqul Umam

**Affiliations:** ^1^Department of Physics, Faculty of Mathematics and Natural Sciences, IPB University, Bogor 16680, Indonesia; ^2^Pusat Studi Sawit, IPB University, Bogor 16143, Indonesia; ^3^Graduate School of Science and Technology, Kwansei Gakuin University, Sanda, Japan

## Abstract

This research aims to examine the characteristics of bulletproof vests from corncob oil palm empty fruit bunch (COPEFB) biocomposite, where mechanical, electrical, and physical resistance tests have been successfully conducted. The variations in the diameter of the twisted thread used to make the basic material for bulletproof vests include 1 mm, 3 mm, 6 mm, and 10 mm, which were tested for their mechanical, electrical, and physical properties. To identify which biocomposite is good at damping bullets, an impact and a firing test were carried out to determine the kinetic energy and the depth of the bullet, respectively. The results showed that the impact value improved with an increase in the diameter of the twisted yarn used. The largest and the lowest impact values were 1.157 kJ and 0.277 kJ on the epoxy sample with a twisted thread diameter of 10 mm and 1 mm, respectively. It was also discovered that the biocomposite samples made from 6 mm to 10 mm twisted threads were the best samples, impermeable to bullets. This was due to the excess natural fiber content which improved the flexibility and absorption of kinetic energy from the high rate of projectile bullets. According to the results of the firing test, some samples are translucent, while others cannot be penetrated by bullet projectiles. The projectile went inside, and the composite was damaged. All the high filler loading samples were translucent to bullets, while some of the low loading samples were translucent and impermeable to bullets. Based on these results, biocomposite samples made of 6 mm and 10 mm twisted yarn are the best samples that are impermeable to bullets.

## 1. Introduction

Indonesia is one of the countries with the largest oil palm plantations in the world. From the calculation survey in 2016, the area for plantations reached 11.67 million hectares [1], which makes Indonesia one of the largest CPO (crude palm oil) exporting countries [2]. In every step of processing and making CPO, not all oil palm bunches have fruit [3]; therefore, these empty bunches usually become unused waste [4].

The use of oil palm waste in various investigations has been carried out since 3 years ago, according to the number of publications on Scopus data with the keywords “Corncob Oil Palm Empty Fruit Bunch (COPEFB)” [5]. However, COPEFB is one of the solid wastes that are not optimally used. This is because several wastes are burned due to insufficient economic value. According to Nikmatin et al. [6], it was discovered that COPEFB waste can be processed and made into new materials through technological breakthroughs and innovations, namely biocomposite material engineering.

Biocomposite is an engineering material composed of more than two materials with different properties [7]. Generally, biocomposites usually have one of the materials that come from nature because the matrix and natural fibers (biofiber) are used as reinforcement in the properties of the material [8]. The main essence of materials engineering is to create new materials with properties superior to the constituents of the previous ones.

Based on a previous report, biofiber is one of the products produced from COPEFB processing [9]. It has flame retardant and mechanical properties that can absorb impact energy through high-impact strength [10]. Nikmatin et al. [3] also recorded that the strength of natural fibers derived from COPEFB depends on the cellulose content and the spiral angle formed between the microfibrillar bonds in the second layer of the cell wall as well as the fiber axis [11]. Natural fibers can be used as fillers for biocomposites that are environmentally friendly [12], have better advantages than synthetic fibers, are easy to process, have a low density of 0.29 ± 0.06 g/cm^3^, and are degradable. Furthermore, the content of biofiber from COPEFB consists of water 8.09 ± 0.09%, ash 2.43 ± 0.03%, pentosan 31.2 ± 0.09%, extractive 7.14 ± 0.09%, lignin 21.75 ± 0.23%, holocellulose 76.09 ± 0.81%, and alpha cellulose 47.50 ± 0.39%. The solubility level of biofiber has a value of 4.41 ± 0.13% in cold water, as well as 5.35 ± 0.18% and 27.17 ± 0.16%, in hot water and NaOH 1%, respectively [3]. These results showed how to improve the use of biofiber from COPEFB waste, specifically as a material for making bulletproof vests.

A bulletproof vest is one of the equipment for self-defense and self-protection by armed forces soldiers. It is usually used as a protector of important organs on the chest, abdomen, back, etc. The bulletproof vest has the main function of resisting penetration and reducing the impact energy generated by projectiles to reduce injuries. When there is a collision between the bullet and the bulletproof vest, the kinetic energy from the bullet is absorbed and distributed over the area of the bulletproof plate, and the remaining energy is transmitted to the soldier's body [13]. In their use, bulletproof vests are expected to dampen and stop projectiles (bullets) by spreading kinetic energy. This is to ensure that the kinetic energy due to ballistic loads on bullets passed on to the user is reduced or lost [14]. In reality, the bulletproof that has been used by the soldier/police (user) and the energy received by the user still cause physical trauma such as bruises, swelling, and other internal injuries [15]. The bulletproof armor is also large enough to reduce the user's movement (mobility). This makes it necessary to search for alternative materials to replace conventional bulletproof materials whose performance is relatively the same in resisting projectile impact energy. Generally, the raw materials for making this armor are Kevlar, metal, and ceramic composites which are imported materials [16].

This research aims to optimize COPEFB waste into a product with economic value and make bulletproof vests with better properties compared to synthetic or previous products. Furthermore, the use of biocomposites from the waste is expected to support environmentally friendly programs by reducing the negative impact of COPEFB waste accumulation.

## 2. Theories

Until now, the industrial world has still used synthetic fibers as reinforcement for composite materials. Synthetic fibers are used in the manufacture of various products such as aircraft, ship hulls, wind turbine blades, car bodies, and others. Of the various types of fibers, glass fiber is the most widely used synthetic fiber. The typical properties of polymeric materials in general are that they can be molded easily at relatively low temperatures; the materials can be printed by injection, pressing, extrusion, and so on, which causes their manufacturing costs to be lower than ceramic materials. In addition, products from polymer materials are light and strong [17]. The specific gravity of polymers is low when compared to metals and ceramics, which is 1.0 to 1.7 gram/cm³, which makes things strong and light. On the other hand, polymer materials also have good resistance to water and chemicals. The selection of a good material will produce a product that has excellent properties but is less resistant to solvents. Generally, polymers can dissolve in certain solvents except for some special materials such as polytetrafluoroethylene. If a polymer has insoluble properties, it will usually crack easily due to continuous contact with solvents and the presence of stress.

The development of natural polymer inventions is aimed at an application in relation to replacing the role of synthetic polymers which tend to be difficult to undergo biodegradation. Recently developed by Nikmatin et al. [6]; palm oil bunch waste is one of the various types of organisms that are useful as natural polymers. The use of polymers depends on their properties, and these properties are determined by their structure and molecular mass. The three main factors (in terms of structure) that determine polymer properties are chemical composition, chain pattern, and the alignment of polymer chains in the final product. These factors, among others, determine the melting point, strength, flexibility, solubility, and reaction of the polymer to heat, while the mass of the polymer molecule determines the solubility of the polymer, printability, and viscosity of the polymer solution (melting). An understanding of the relationship between these properties and structures, as well as the ability to build polymer structures according to the desired properties, is an important capital for the development of the polymer industry [18].

Based on the constituent monomers, polymers are large molecules and are generally represented by depicting only one chain [19]. A chain described above must include at least one complete repeat unit. Like the structure of broken cellulose molecules. Cellulose is a polymer found in the cell walls of plants such as wood, branches, and leaves. Cellulose is what makes the structures of wood, branches, and leaves strong. With their properties, polymers can be divided into three general groups, namely, elastomers, fibers, and plastics. The characteristic of an elastomer is its ability to be stretched under stress (stretched) and return to its original shape when the pressure is reduced (elastic). Examples of elastomers include rubber (natural and synthetic) and silicone. Fibers are polymers that have high tensile strength properties along their axes. Fibers are thread-like polymers that can be woven into fabrics. Cotton, wool, and silk are examples of natural fibers. Some synthetic fibers, such as nylon, orlon, and dacron, have additional beneficial properties, namely, increased tensile strength; lighter, low moisture absorption; resistance to moths, mildew, rot, and mildew; and not wrinkled. Plastics have properties intermediate between those of elastomers and fibers, with varying properties at room temperature. Examples are polystyrene (PS) and polypropylene (PP). Polystyrene is stiff and brittle, whereas polypropylene is very hard, impact-resistant, tear-resistant, and flexes in thin sheet form.

Based on their properties, polymers can be classified into four properties, namely, chemical, physical, mechanical, and thermal properties. The following is a classification of polymers based on chemical, physical, mechanical, and thermal properties [20].

### 2.1. Chemical Properties

The attractive forces between the polymer chains play a major role in the properties of the polymer. Because polymer chains are very long, the forces between the chains are many times greater than the usual attractions between molecules. Different side groups can result in ionic or hydrogen bonding of the polymer in the same chain. The stronger the force, the higher the tensile strength, melting point, and crystallinity level. The intermolecular forces in the polymer can be affected by the dipole in the monomer units. Polymers containing amide or carbonyl groups can form hydrogen bonds between adjacent chains. The positively charged hydrogen atom in the N-H group will be strongly attracted to the negatively charged oxygen in the C=O group. This strong hydrogen bond will result in an increase in tensile strength and melting point, for example, in polymers containing urethane or urea. Polyester has dipole-dipole bonds between the oxygen atoms on C=O and the hydrogen atoms on the C-H groups. Dipole bonds are not as strong as hydrogen bonds, so the melting point of polyester is lower, but it has high flexibility.

### 2.2. Physical Properties

Several factors affect the physical properties of polymers as follows: (1) Strength and melting points increase with increasing polymer chain length. (2) If the intermolecular forces on the polymer chain are large, the polymer will become strong and difficult to melt. (3) Polymer chains that have many branches have low tensile strength and melt easily. (4) Cross-linking between polymer chains causes a rigid network and forms a hard material. The more cross-links, the stiffer the polymer and the easier it will break. (5) Polymers with irregular structures have low crystallinity and are amorphous (not hard). Meanwhile, polymers with regular structures have high crystallinity, making them stronger and more resistant to chemicals and enzymes.

### 2.3. Mechanical Properties

Strength is one of the mechanical properties of polymers. There are several kinds of strength in polymers, including the following: (1) Tensile Strength is the stress required to break a sample. Tensile strength is important for the polymer to be stretched, for example, fiber must have good tensile strength. (2) Compressive strength is resistance to pressure. Concrete is an example of a material that has good compressive strength. Anything that has to support the weight from below must have good compressive strength. (3) Flexural strength is resistance to bending (flexing). Polymers have flexural strength if they are strong when bent. (4) Impact strength is resistance to stress that comes suddenly. A polymer has impact strength if it is strong enough to be hit suddenly, such as with a hammer. (5) Elongation is one type of deformation. Deformation is a change in size that occurs when the material is subjected to a force. % Elongation is the length of the polymer after being subjected to force (*L*) divided by the length of the sample before being subjected to force (Lo) then multiplied by 100. (6) The modulus is measured by calculating the stress divided by the elongation. The unit for modulus is the same as the unit for strength (N/cm^2^). (7) Toughness is the actual measurement of the energy a material can absorb before it breaks.

### 2.4. Thermal Properties

The characteristic properties of polymeric materials are greatly changed by changes in temperature. This is because if the temperature changes, the movement of molecules due to temperature will change the structure (especially structures with large dimensions). Furthermore, because heat, oxygen, and water together provoke chemical reactions in the molecules, depolymerization, oxidation, hydrolysis, and so on occur at high temperatures. The coefficient of long expansion in films and fibers often decreases due to heat, because when the temperature is increased, the way the molecules aggregate changes due to the thermal motion of the molecules. In addition, the specific heat of polymeric materials is approximately 0.25–0.55 cal/g/°C which is greater than that of metal materials, also greater than that of ceramics. This is because specific heat is the heat used for the thermal movement of molecules in their structures. The thermal conductivity coefficient is an important value for polymer materials due to the heat of printing and use of the product. The mechanism of heat transfer in polymeric materials is also a result of heat propagation from the movement of molecules.

## 3. Method

**Figure 1:**
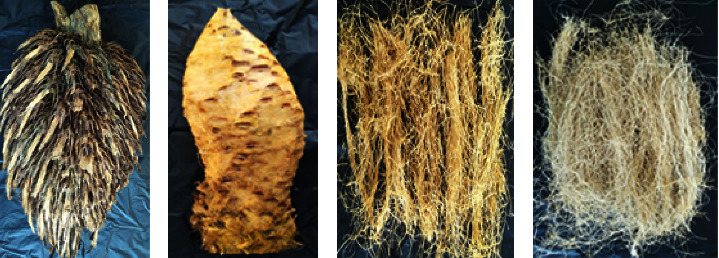
(a) COPEFB, (b) stalk, (c) fiber before washing, and (d) fiber after washing. In the process, COPEFB is converted into stalk by removing spikelets. Furthermore, the stalk is processed into fiber and washed in the final stage.

**Figure 2:**
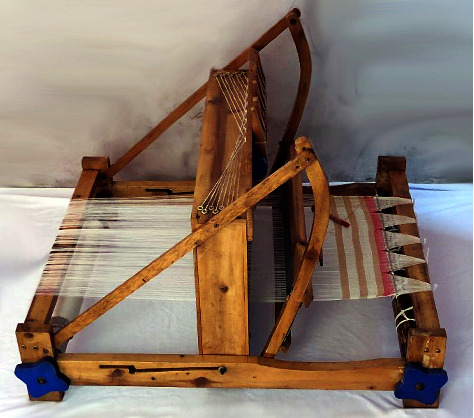
Loom not machine (LNM).

The main material used was COPEFB obtained from PTPN VIII Cikasungka, Bogor Regency, West Java, Indonesia. Kevlar fiber was also purchased in the market as a comparison with the final product. COPEFB preparations become stalks before finally becoming long fibers, as shown in Figure 1. The long fibers were washed before being made them into twisted yarn and sheets of cloth using a loom not machine (LNM), as presented in Figure 2. This was carried out at the Muda Manunggal Alam farmer group around the Jasingga palm oil plantation in Bogor.

**Figure 3:**
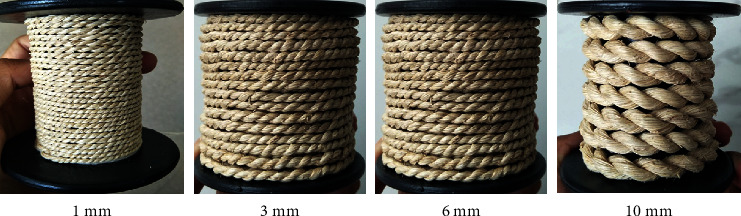
Undyed COPEFB twisted yarn with various diameters (mm).

Twisted yarn is a combination of 5–50 strands of COPEFB filament yarn (biofiber) to provide special strength in certain applications such as ropes, raw materials for fashion creative industries, basic fabrics, and shoe pads. The part of the COPEFB used was the stalk that has been retting until it decomposes and mechanical fibrillation was given into long fibers measuring 20–30 cm, which were dried in the open air and under the sun [6]. The next stage is to combine each strand of long fiber into a continuous unit which is twisted into 4 diameter variations, namely, 1 mm, 3 mm, 6 mm, and 10 mm as shown in Figure 3. Meanwhile, the main equipment used is mechanical milling, a twisting machine, a hot press, and LNM. The process in this research consists of aforementioned six stages.

The first stage is the processing of COPEFB into stalk and retting for 6 days, followed by 3 dynamic cycles of washing in an open system of mechanical fibrillation in situ until long fibers were produced. The COPEFB long fibers were dried in the sun to obtain a uniform and homogeneous moisture content of <7%. Subsequently, 20 strands of fiber were weighed and measured for length, thickness, and ratio and given a tensile force on each strand to calculate its ability to accept deformation until it breaks. The COPEFB long fibers are made of continuous longitudinal twisted yarns by crossing each strand at various diameters of 1 mm, 3 mm, 6 mm, and 10 mm. The twisted yarn was made of cloth and woven using LNM (traditional) at a size of 50 × 50 mm, totaling 100 pieces in each variation of the diameter of the twisted yarn.

The second stage is testing the quality of COPEFB fiber as a substitute for synthetic kevlar fiber. It also includes testing the chemical composition based on the standards of the Technical Association of the Pulp and Paper Industry (TAPPI), namely TAPPI T222 om-88 (1988) with modifications by Dence for lignin, TAPPI T203 OS-61 (1961) for cellulose, TAPPI T9m-54 (1988) for holocellulose, and TAPPI T204 om-88 (1988) for ethanol-benzene extractives. The COPEFB fat content test was measured using a Soxhlet Apparatus with a sample of 3 grams and extraction for 6 hours using 150 ml of fat solvent in form of hexane. Furthermore, the COPEFB fiber density test was carried out using the Archimedes method on physical and mechanical properties.

**Figure 4:**
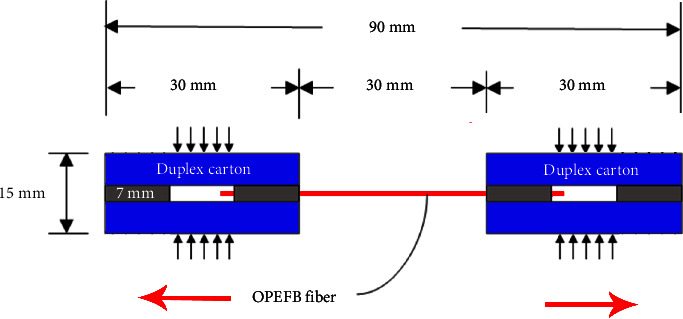
Illustration of ASTM D3379-75 tensile strength test.

The COPEFB long fiber was tested for tensile strength using ASTM D3379-75. COPEFB fibers were taken in long lengths and glued onto duplex cardboard, as shown in Figure 4. Meanwhile, tensile strength is the maximum stress that a material can withstand when it is stretched or pulled before breaking. Generally, tensile strength can be determined by performing a tensile test and noting changes in strain and stress. The maximum tensile strength is the highest point of the stress-strain curve. Based on the results of this test, a graph of the load versus elongation or elongation can be made. The dimension of the tensile strength is the force (*F*) per unit area (*A*) i.e. N/m^2^ or Pascal (Pa). The calculation of load and elongation is formulated in equation:

(1) $$\begin{array}{c} Stress\;\left(\sigma \right)=\frac{F}{A}. \end{array}$$

The strain is the amount of change in length increase caused by loading compared to the length of the initial measuring area (gage length). It is obtained from (2) by changing the length (*l*_2_ − *l*_1_) compared to the original length (*l*_1_).

(2) $$\begin{array}{c} Elongation\;\left(\varepsilon \right)=\frac{l_{2}-l_{1}}{l_{1}}\text{.} \end{array}$$

The third stage is testing the electrical properties in form of conductance/resistance values and the dielectric constant of the biofiber that has been made into twisted yarn. This test was carried out by passing an electric current on twisted threads with diameters of 1 mm, 3 mm, 6 mm, and 10 mm. The electrical properties test is carried out to determine whether the twisted yarn made of COPEFB biofiber is a conductor or an insulator. In the analysis, Equations (3) [21] and (4) [22] were used to determine the electrical properties of twisted yarn.

(3) $$\begin{array}{c} C=k\frac{\in_{0}A}{d}\text{,} \end{array}$$

with value *K*=∈/∈_0_; *d* is the distance between materials (m), *A* is the cross-sectional area (m^2^), *C* is the capacitance (farad), *k* is the dielectric constant of air (1,00054), *K* is the dielectric constant of the material, ∈_0_ is the permittivity of the vacuum (8,854 × 10^–12^ F/m), and ∈ is the permittivity of the material (F/m).

(4) $$\begin{array}{c} \sigma =\frac{L}{A}\times \frac{1}{R}=\frac{L}{A}G\text{,} \end{array}$$

with value *σ*=1/*ρ*; *L* is the thickness of the material (cm), *A* is the area of the base of the material *R* is resistance (ohms), *G* is the conductance (siemens), *σ* is conductivity (S/cm), and *ρ* is resistivity (ohm meter).

The fourth stage is the manufacture of a biocomposite Sandwich (bulletproof sheet) by mixing polymer (epoxy or polyester) and a concentration of COPEFB biofiber fibers. The biocomposite sheets were arranged with various diameters in a Sandwich based on an angle of 0, 45, and 90 degrees. Each sheet was coated with adhesive and a mixture of polymers (epoxy or polyester). The pile of fabrics was varied from 10 sheets, 15 sheets, to 20 sheets and pressed at a temperature of 60°C until the final thickness reached 2 cm.

**Figure 5:**
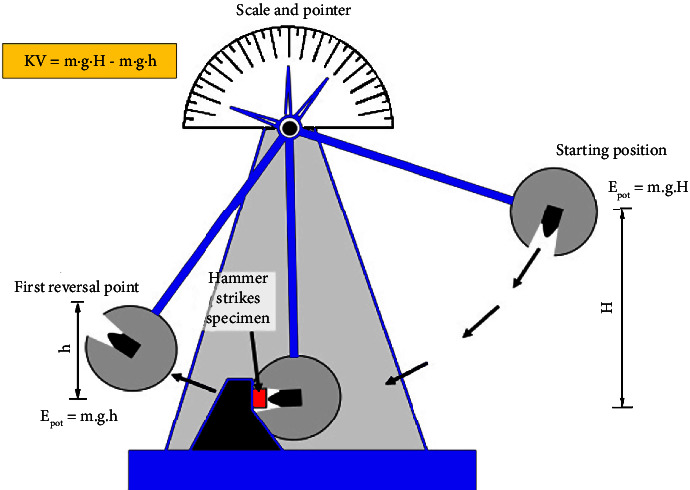
Illustration of ballistic impact strength test (ASTM D-256A) using notched izod impact strength.

The fifth stage is impact strength (ASTM D-256A) or ballistic testing using Notched izod impact strength. Mechanical tests were also carried out on biofibers that were made into sheets and arranged or can be referred to as Sandwich biocomposites. Biocomposite testing (bulletproof sheet) was carried out using impact strength (ASTM D-256A) or ballistic testing using Notched izod impact strength as shown in Figure 5. Notched izod impact strength is a measure of the impact resistance of a material. Its working principle is by observing the movement of the pendulum that shifts the needle on the tool and the impact energy value is observed directly from the scale indicated by the needle. This test used a tool with an impact speed of 3.5 m/s, a pendulum arm length of 0.322 m, and a mass of 0.5 kg. Mechanical impact strength testing is a test to measure the impact resistance of a material [23]. This test also aims to identify the strength of the material against deformation from the outside.

During the impact test, there is a rapid impact, and the kinetic energy of the pendulum strikes the specimen, making the energy to be absorbed by the biocomposite material. The pendulum is raised to a certain height (constant potential energy) and released. Subsequently, the pendulum swings down hitting the sample and breaking the specimen. The energy absorbed by the sample is calculated from the height of the arm swing after touching the sample. The sample is made a notch to determine the energy sensitivity and impact strength [24]. The impact strength value is calculated from the impact energy data from the tool divided by the thickness of the test specimen.

**Figure 6:**
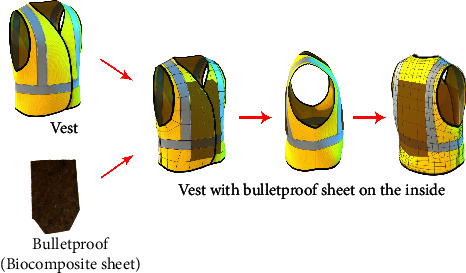
Illustration of a vest that has been installed with a bulletproof sheet (the installation is placed in the vest on the front and back of the vest).

The sixth stage is a shooting test (horizontally) using a 9 × 19 mm glock pistol with a 12.25 gram MU1-TJ bullet at a shooting range of 30 meters. In this test shot, the vest was coated with bulletproof sheets (biocomposite Sandwich) on the front and back as shown in Figure 6. The shooting test was carried out at the 2 coconut shooting ranges in Depok, West Java, and Pindad Malang, East Java. Meanwhile, data supporting material structure testing was also carried out using X-ray Diffraction (XRD) to analyze crystallography and Fourier Transform Infrared Spectroscopy (FTIR) for molecular functional group analysis. Differential Scanning Calorimetry (DSC) was also used to study phase transitions such as melting, transition temperature glass, decomposition, exothermic, and endothermic.

Specimen (Bulletproof) is a prototype biocomposite that has been made and consists of COPEFB fiber and polymer. The shooting test was carried out by setting the position of the specimen on the pedestal between the right and left sides with the shooter's position. This was followed by the use of a weapon according to the testing standards in this research. The shooter's position stood parallel to the biocomposite specimen at a distance of 30 m and the specimen was observed by the pattern of damage and the depth of penetration that occurs.

## 4. Result and Discussion

### 4.1. Chemical Characteristics of Biofiber from Corncob Oil Palm Empty Fruit Bunch (COPEFB)

In this research, the twisted yarn yield (long fiber finish) of COPEFB had an average length of 144.70 ± 25.92 mm and a fiber diameter of 0.29 ± 0.07 mm. These results were obtained after the preparation of raw materials, the cooking process, and bleaching. The cooking and bleaching process increased the efficiency of mechanical defibrillation in the extraction process of nonfibrillated cellulose, which was based on removing impurities and destroying the cell wall structure [25]. The results of the analysis of COPEFB fiber (biofiber) mechanically and retting without chemicals contain a chemical composition of alpha cellulose 46.62 ± 0.11%, lignin 23.17 ± 0.38%, ash content 3.35 ± 0.01%, and extractive 7.60 ± 0.17%. Meanwhile, the solubility level of biofiber has a value of 7.74 ± 0.08% in cold water, 8.19 ± 0.16% in hot water, and 26.91 ± 0.23% in NaOH 1%, as shown in Table 1.

The results of density analysis using Archimedes' law or method have an average value of 0.60 g/cm^3^ with a standard deviation of 0.08 as presented in Table 2. *V*_*f*_ is the volume of the fluid and *V*_*s*_ is the volume of the sample.

### 4.2. Analysis of the Mechanical Properties of Twisted Yarn Using the Tensile Strength Method ASTM D3379-75

Impact strength testing is carried out by measuring the load resistance to shock loads. The test is usually conducted to measure the amount of energy absorbed by a material until it breaks. In previous research, the variation of polymer and the concentration of the fiber filling the biocomposite can affect the impact strength of a biofiber [9]. Therefore, the higher the impact value, the more flexible the material. This showed that the amount of fiber used affects the flexibility of the biocomposite material because it becomes more resilient and ductile [26]. The differences in impact values can be caused by variations in fiber concentration in the composite because the fiber withstands the energy from the impact. Fibers that do not fill the entire space in the composite become inflexible and have a small impact value [11].

Mechanical testing of twisted yarn was carried out according to textile standards at the Textile Center of the Ministry of Industry Bandung, West Java, with 10 repetitions of each sample. Tensile strength shows the ability of the material to accept tensile forces in the vertical direction. Meanwhile, strain is the ability of a material to stretch until it breaks. In the results of the mechanical properties test with diameter variations as shown in Table 3, it was discovered that the tensile strength and elongation improve with an increase in fiber diameter. There is currently an increase in the use of COPEFB fiber (biofiber), making the diameter bigger to give force and energy to the material in accepting external loads or forces.

### 4.3. Analysis of the Electrical Properties of Twisted Yarn Based on the Value of Conductivity and Dielectric Constant

Electrical properties are the identification of the classification of electrical materials based on conductivity and dielectric constant as presented in Tables 4 and 5. In the electrical properties test, only twisted yarns formed in diameters of 1 mm and 3 mm were detected, while 6 mm and 10 mm were not found. The test was carried out to determine whether it is a conductor or an insulator. The results showed that the twisted yarn with a diameter of 1 mm and 3 mm has a high resistance value, therefore, the twisted yarn from COPEFB is an insulator.

### 4.4. Analysis of Mechanical Properties of Biocomposite (Bulletproof) by Testing Ballistic Impact Strength (ASTM D-256A) Using Notched Izod Impact Strength

The ballistic impact strength test (ASTM D-256A) was also carried out to measure the load resistance to shock loads. Moreover, it is the absorption of potential energy or a load that swings from a certain height and strikes the object being tested to pass through deformation (change). Therefore, impact testing is carried out to measure the amount of energy absorbed by the biocomposite [27]. The polymer and the concentration of the fiber that fills the biocomposite affect the impact strength of the biocomposite as shown in Table 6 below.

Based on Table 6, the impact value increases with the high diameter of the twisted yarn. The result showed that the largest and lowest impact values are 1.157 kJ and 0.277 kJ on the epoxy sample with a twisted thread diameter of 10 mm and 1 mm, respectively. This indicated that the higher the impact value, the more flexible the material. Therefore, it can be assumed that the amount of fiber used affects the flexibility of the biocomposite material, which becomes more resilient and ductile [28].

The difference in impact value can be caused by the variation in the diameter of the twisted yarn in each biocomposite. The large diameter of the twisted yarn affected the impact value because the fiber can withstand the energy of the impact. Meanwhile, fibers that do not fill the entire space in the biocomposite become inflexible and have a small impact value. The largest impact value was in the sample with a diameter of 10 mm twisted thread on epoxy and polyester polymers. This showed that the addition of fiber affected the impact strength of a material. Impact strength is the energy that can be absorbed by a material due to the sudden loading of the force until damaged. The impact strength value indicates that COPEFB fiber has a good ability to absorb a suddenly applied force. This indicated that the more fiber used as filler in the biocomposite, the greater the force absorbed. This was caused by the influence of the distribution of forces on the filler. Furthermore, the filler composition affected the force absorbed by each filler particle to increase the resistance of the material to a suddenly applied force [4]. The diameter of the twisted thread also influenced the resistance of the material to withstand the applied force.

### 4.5. Analysis of the Mechanical Properties of the Biocomposite by Firing Test (Horizontally) Using a 9 × 19 mm Glock Pistol with a 12.25 Gram MU1-TJ Bullet at a Shooting Range of 30 Meters

The mechanical analysis was a biocomposite test using a 9 × 19 mm glock gun with a 12.25 Gram MU1-TJ bullet at a shooting range of 30 meters. Based on the results of the firing test, some samples are translucent, while others cannot be penetrated by bullet projectiles. The projectile went inside and the composite was damaged. All of the high filler loading samples were translucent to bullets, while the low filler loading samples were translucent and some were impermeable to bullets, as shown in Table 7.

Table 7 shows that samples C and D are the best because they are not penetrated by bullets. This was due to the higher fiber content, where samples C and D used COPEFB twisted yarn diameters of 6 mm and 10 mm, respectively. COPEFB fiber can withstand a collision caused by straighteners that have flexibility because they absorb energy from the bullet projectile to avoid penetration into the biocomposite. It was also discovered that the average value of the shooting distance decreases with the diameter of the twisted thread used. This is because the bullet was considered as a force against the biocomposite with the highest diameter of the twisted thread having a smaller average depth of shot.

Moreover, samples G and H are the best because they are not penetrated by bullets. This was due to the higher fiber content, where in sample G, the diameter of the 6 mm COPEFB twisted yarn had an average depth of shot of 3 mm. Sample H with a 10 mm diameter of the COPEFB twisted yarn had an average depth of shot of 2.96 mm. Based on these results, the average depth of shot decreases as the concentration of the fiber used increases. This was because the fiber can absorb the force exerted by the bullet on the biocomposite for the biocomposite with the highest twisted thread diameter to have a higher average depth of shot [15]. Fiber is the main element in the composite, which determines the characteristics of the composite material such as stiffness, strength, and mechanical properties. According to Sahari and Sapuan [27]; fiber withstands most of the forces acting on the composite material, while the matrix act as a protector and binder for the fiber to work properly. On the other hand, these results also correlate with previously research conducted by Nikmatin et al. [4] entitled “Kinematics and Dynamics of the Ballistic Impact Behavior for an Oil Palm Empty Fruit Bunch Fiber Reinforced Bio-Composite,” it is known that The epoxy biocomposite was able to hold a projectile more successfully than the polyester biocomposite with the curve of the decrease in velocity for both of the resins was exponentially distributed. An 18% epoxy biocomposite was able to more successfully stop the projectile at a penetrative depth of 2.14 mm and was able to absorb all the kinetic energy generated (408 J).

## 5. Conclusion

Bulletproof vests from COPEFB biocomposites were made with various diameters, namely 1 mm, 3 mm, 6 mm, and 10 mm. The test analysis was carried out to determine the mechanical, electrical, and physical resistance properties. The results of the analysis of COPEFB fiber (biofiber) mechanically and retting without chemicals contain a composition of alpha cellulose 46.62 ± 0.11%, lignin 23.17 ± 0.38%, ash content 3.35 ± 0.01% and extractive 7.60 ± 0.17%. Meanwhile, the solubility level of biofiber has a value of 7.74 ± 0.08% in cold water, 8.19 ± 0.16% in hot water, and 26.91 ± 0.23% at 1% NaOH. Based on the mechanical properties test with variations in the diameter of 1 mm, 3 mm, 6 mm, and 10 mm, it was discovered that the tensile strength and elongation increased along with the high fiber diameter. Due to the increase in the use of biofiber, the diameter is also improved to give force and energy to the material in accepting external loads or forces. Meanwhile, in the electrical properties test, only twisted threads formed in diameters of 1 mm and 3 mm were detected, while diameters of 6 mm and 10 mm were not detected. The electrical properties test was carried out to determine whether the material is a conductor or an insulator. The results showed that the twisted yarn with a diameter of 1 mm and 3 mm has a high resistance value, indicating it is an insulator. Moreover, ballistic impact strength testing (ASTM D-256A) was also carried out to measure the load resistance to shock loads. According to the results of the firing test, some samples are translucent, while others cannot be penetrated by bullet projectiles. The projectile went inside and the composite was damaged. All the high filler loading samples were translucent to bullets, while some of the low samples were translucent and impermeable to bullets. Based on these results, biocomposite samples made of 6 mm and 10 mm twisted yarn are the best samples that are impermeable to bullets.

## Figures and Tables

**Table 1:** The results of the analysis of the chemical characteristics of the fiber (biofiber) from COPEFB.

No	Parameter	Analysis results (%)	Test method	Research of literature (%) [3]
		COPEFB		
1	Water	7.66 ± 0.04	SNI 087070	<10
2	Ash	3.35 ± 0.01	SNI ISO 1762	0.3–1.5
3	Pentosan	27.52 ± 0.11	SNI 01-1561	19–25
4	Extractive	7.60 ± 0.17	SNI 8401	2–5
5	Lignin	23.17 ± 0.38	SNI 8429	20–33
6	Holocellulose	71.59 ± 0.02	Metode wise^*∗*^	55.40–76.68
7	Alpha cellulose	46.62 ± 0.11	ASTM D 1103	40–45
8	Cold water solubility	7.74 ± 0.08	SNI 01-1305	1.3–3.5
9	Hot water solubility	8.19 ± 0.16	SNI 01-1305	1.38–7.37
10	1% NaOH solubility	26.91 ± 0.23	SNI 14-1838	17.1–23.4

Source: data of this research.

**Table 2:** The results of the biofiber density test using Archimedes hukum law.

No	Corncob oil palm empty fruit bunch (COPEFB)				
	Mass (gram)	Initial *V*_*f*_ (mL)	End *V*_*f*_ (mL)	*V*_*s*_ (mL)	Density (g/cm^3^)
1	0.5	40	41	1	0.50
2	1	40	42	2	0.50
3	1.5	40	43	3	0.50
4	2	40	43	3	0.67
5	2.5	40	44	4	0.63
Average					0.60
Standard deviation					0.08

Source: data of this research.

**Table 3:** Mechanical properties of twisted yarn at various diameters.

Mechanical properties	Variation of diameter twisted yarn (mm)			SNI test standard [3]
	1	3	6	
Tensile (kgf)	3.55 ± 0.95	13.35 ± 1.65	56.75 ± 7.68	7650-2010
Strain (%)	20.13 ± 5.76	45.57 ± 7.89	65.98 ± 4.19	7650-2010
Elongation (mm)	50.42 ± 14.40	113.98 ± 19.79	165.13 ± 10.51	7650-2010

Source: data of this research.

**Table 4:** Electrical conductivity of twisted yarn.

No	Sample	*d*(cm)	*A*(cm^2^)	*R*(Ω)	*G*(S)	*ơ*(S/cm)	*ơ*average	Dev
1	D1 (1 mm)	0.048	0.03595	8.5*E* + 07	1.2*E* − 08	2*E* − 08	2*E* − 08	9.96*E* − 10
		0.048	0.03595	9.6*E* + 07	1.0*E* − 08	1*E* − 08		
		0.048	0.03595	8.6*E* + 07	1.2*E* − 08	2*E* − 08		

2	D2 (3 mm)	0.094	0.20820	2.0*E* + 08	5.1*E* − 09	2*E* − 09	2*E* − 09	1.55*E* − 10
		0.094	0.20820	1.8*E* + 08	5.7*E* − 09	3*E* − 09		
		0.094	0.20820	1.8*E* + 08	5.6*E* − 09	3*E* − 09		

Source: data of this research.

**Table 5:** Yarn dielectric constant.

No	Sample	*d*(m)	*A*(m^2^)	*C*(F)	*ɛ*ingredient (F/m)	*ɛ*air (F/m)	*k*	*k*
1	D1 (1 mm)	0.00048	1.38*E* − 05	8*E* − 13	2.77*E* − 11	8.85*E* − 12	3.1	3.9 ± 1.03
		0.00048	1.38*E* − 05	9*E* − 13	3.12*E* − 11	8.85*E* − 12	3.5	
		0.00048	1.38*E* − 05	1.3*E* − 12	4.51*E* − 11	8.85*E* − 12	5.1	

2	D2 (3 mm)	0.00094	3.32*E* − 05	1.9*E* − 12	5.41*E* − 11	8.85*E* − 12	6.1	6.4 ± 0.32
		0.00094	3.32*E* − 05	2.1*E* − 12	5.99*E* − 11	8.85*E* − 12	6.8	
		0.00094	3.32*E* − 05	2*E* − 12	5.70*E* − 11	8.85*E* − 12	6.4	

Source: data of this research.

**Table 6:** Results of ballistic impact strength (ASTM D-256A) testing using notched izod impact strength on biocomposites.

COPEFB twisted thread diameter (mm)	Polymer	Kinetic energy (kJ)
1	Epoxy	0.277
3	Epoxy	0.320
6	Epoxy	0.585
10	Epoxy	1.157

1	Polyester	0.261
3	Polyester	0.672
6	Polyester	0.767
10	Polyester	1.091

Source: data of this research.

**Table 7:** Ballistic testing of bulletproof material samples with varying concentrations and polymer mixtures (epoxy and polyester).

Sample	COPEFB and polymer twisted yarn diameter	Depth of bullet through bulletproof biocomposite (mm)						Average (mm)
		Test 1	Test 2	Test 3	Test 4	Test 5	Test 6	
A	1 mm + epoxy	Perforated	—	—	—	—	—	—
B	3 mm + epoxy	Perforated	—	—	—	—	—	—
C	6 mm + epoxy	2.20	2.45	2.22	2.50	2.21	2.20	2.29 ± 0.14
D	10 mm + epoxy	2.23	2.12	2.14	2.02	2.21	2.15	2.14 ± 0.07
E	1 mm + polyester	Perforated	—	—	—	—	—	—
F	3 mm + polyester	Perforated	—	—	—	—	—	—
G	6 mm + polyester	2.94	3.10	3.00	3.13	2.88	2.97	3.00 ± 0.13
H	10 mm + polyester	2.71	3.00	3.00	2.94	3.12	3.00	2.96 ± 0.09

Source: data of this research.

## Data Availability

The data used to support the findings of this study are available from the corresponding author upon reasonable request.
